# Effect of Ni and Nb Elements on Corrosion Resistance and Behavior of TC4 Alloy in Hydrochloric Acid

**DOI:** 10.3390/ma18020246

**Published:** 2025-01-08

**Authors:** Kaikai Xiao, Jinpeng Ge, Yongqiang Zhang, Jian Wang, Weizhong Feng, Xingyu Ou-Yang, Yang Yu, Wenjun Ye, Songxiao Hui

**Affiliations:** 1 Baoji Titanium Industry Company Limited, Baoji 600456, Chinazhangyongqiang@baoti.com (Y.Z.);; 2 BAOTI Group Company Limited, Baoji 721014, China; 3 Soleras Advanced Coatings BV, 201 JinShan Road, Jiangyin 214437, China; 4 GRIMAT Engineering Institute Company Limited, Beijing 101407, Chinahuisx@grinm.com (S.H.)

**Keywords:** TC4 alloys, acid corrosion resistance, electrochemistry, passive film

## Abstract

Due to the development of the petroleum industry, more severe mining conditions put forward higher corrosion resistance requirements for materials. In this paper, the corrosion resistance and corrosion behavior of four TC4-xNi-yNb (x, y = 0, 0.5) alloys were investigated in a 1 mol/L HCl solution through microscopic characterization, electrochemical tests and corrosion weight loss testing. The results demonstrated that the addition of Ni and Nb elements could improve the corrosion resistance of TC4 alloy to varying degrees. The addition of niobium formed niobium oxide in the passive film, while the addition of nickel thickened the passive film without formation of nickel oxides. The improvement of corrosion resistance of TC4 by nickel is more significant. Finally, a new highly corrosion resistant alloy TC4-0.5Ni-0.5Nb is preferred.

## 1. Introduction

The demanding high-temperature, high-pressure, acidic corrosive environment and complex alternating loads in actual working conditions place higher demands on the corrosion resistance and mechanical performance of oil well pipe materials as the depth of oil and gas exploration and extraction continues to increase. The trend in the selection of oil well pipe materials is primarily focused on high corrosion-resistant alloys [1,2,3]. Titanium alloys possess high specific strength, excellent corrosion resistance, low elastic modulus, and non-magnetic properties and exhibit good resistance to CO_2_ corrosion and sulfide stress corrosion, providing new solutions due to their unique combination of properties. Furthermore, titanium resources are abundant, and the cost is lower than nickel-based alloys [4,5,6]. Titanium alloys with excellent mechanical properties and corrosion resistance have become the ideal materials for high-end oil well tubing [7].

The TC4 titanium alloy, the most mature and widely used system, exhibits significant passivation behavior in corrosive downhole environments containing CO_2_, H_2_S, and Cl^−^. However, TC4 is prone to severe crevice corrosion and has a higher tendency to stress corrosion cracking when temperatures are higher [8,9,10]. LYU et al. [11] investigated the corrosion resistance of TC4 alloy in geothermal water/CO_2_ and completion fluid/CO_2_ environments at different temperatures using high-temperature and high-pressure corrosion test equipment. The results indicate that TC4 alloy exhibits excellent resistance to stress corrosion cracking in low temperature and corrosion environments. However, significant pitting and selective corrosion occurred in the presence of completion fluid/CO_2_ corrosion at 180 °C. Liang et al. [12] conducted a study on the corrosion resistance of TC4 alloy in a simulated environment with CO_2_, H_2_S, and Cl^−^ in formation water. The results show that the alloy’s polarization resistance decreases with increasing temperature, leading to a reduction in corrosion resistance.

Researchers have modified the TC4 alloy by adding high-corrosion-resistant alloying elements to enhance the applicability of TC4 alloy in the field of oil and gas development. The addition of platinum group metals can significantly enhance the resistance to crevice corrosion and stress corrosion capability of titanium alloys in reducing acidic environments. This is because the dissolution of platinum group metals results in the deposition of fine, dispersed metal particles on the surface of the titanium alloy. These metal particles elevate the electrode potential of the passivation film on the metal surface and attract a significant number of hydrogen ions. This process mitigates the increase in acidity within crevices, effectively reducing the alloy’s susceptibility to crevice corrosion [12,13,14,15]. Ueda et al. [16] studied the high-temperature corrosion behavior of titanium alloys containing Mo and Pd in environments containing S^−^, H_2_S^−^, CO_2_, and Cl^−^. The addition of Pd or an increase in Mo content results in higher stability of TiO_2_ films over a broader pH range. Additionally, both Pd and Mo can enhance the resistance to stress corrosion cracking caused by the active path in the alloy, making them highly effective in improving stress corrosion cracking resistance. Schutz et al. [17] has designed a relatively low-cost, highly corrosion-resistant TC4-0.1Ru alloy which exhibits excellent corrosion resistance in high-temperature, acidic oil and gas fields. TC4-0.1Ru alloy also enhances the high-temperature corrosion resistance to chlorides up to 300 °C, improves resistance to stress corrosion cracking in high-temperature Cl^−^ containing media and mitigates stress and crevice corrosion in the alloy. Currently, alloying elements Mo, Cr, Nb, Zr, Ni are primarily added to TC4 alloys for corrosion resistance modification [18]. These alloying elements contribute to increasing the alloy’s electrode potential and passivation ability. They also form oxides within the passive film, strengthening the passivation layer on titanium alloys, thus enhancing their resistance to stress corrosion cracking. However, there are few reports on the effect of the combination of two elements on the corrosion resistance of TC4 alloys in acidic environments. A combination of alloying elements may result in better corrosion resistance.

This article focused on the study of TC4 alloys with different Ni and Nb element additions including five different alloy compositions: TC4, TC4-0.5Ni, TC4-0.5Nb and TC4-0.5Ni-0.5Nb. This research utilized a 1 mol/L HCl solution as the corrosive medium and conduct the following experiments at room temperature: electrochemical testing, immersion corrosion experiments, weight loss measurements post-immersion, morphology observation, analysis of corrosion products, and examination of surface passivation films. This research aimed to study the corrosion behavior of alloys with different Ni and Nb element additions in hydrochloric acid solutions and analyze the mechanisms of how Ni and Nb elements enhance the corrosion resistance of the alloy to select alloy compositions with excellent corrosion resistance.

## 2. Materials and Methods

### 2.1. Materials

Four different alloy compositions were employed, namely TC4-xNi-yNb (x, y = 0, 0.5). Ingots with dimensions of Φ210 mm × 560 mm were prepared by twice vacuum self-consuming melting using Grade 0 sponge titanium, electrolytic nickel, Al-55V alloy, Ti-Nb intermediate alloy, TiO_2_ and aluminum beans. Then, the ingots were forged into a block, hot-rolled into plates with deformation of 35% after holding at 900 °C for 1 h, followed by air cooling. Finally, annealing was performed at 500 °C for one hour to reduce residual stress. The chemical compositions analysis of the plates is shown in Table 1. Electrochemical samples with dimensions of 10 mm × 10 mm × 2 mm were encapsulated and polished with 1000-grit sandpaper. The dimensions of the test samples for immersion corrosion were 30 mm × 20 mm × 2 mm. All surfaces of the samples were polished to a 2000-grit finish and further polished to a mirror surface. Subsequently, samples were subjected to ultrasonic cleaning for 3 min in deionized water and alcohol, followed by drying with cold air.

### 2.2. Microstructural Analysis

Observation of the microstructural and corrosion morphology of the alloys was conducted using a JSM-F100 scanning electron microscope (SEM) (Tokyo, Japan). An AXIS Ultra DLD multifunctional X-ray photoelectron spectrometer (XPS) (Manchester, UK) was employed to conduct a full-spectrum scan of the composition and elemental valence states of the passivation film on the alloy surface. The obtained spectra were analyzed using Avantage 6.6.6 software with calibration of all spectral binding energies performed using surface-contaminating carbon.

### 2.3. Electrochemical Testing

Electrochemical measurements were conducted on the Versa STA 4 electrochemical workstation (Oak Ridge, TN, USA). A 1 mol/L HCl solution was chosen as the corrosive solution and test exposure area is 1 cm^2^. The auxiliary electrode was a platinum electrode, the reference electrode was a saturated calomel electrode (SCE) and the working electrodes were the test samples. The open circuit potential (OCP) test duration was 3000 s. The frequency parameters for electrochemical impedance spectroscopy (EIS) ranged from 0.01 Hz to 105 Hz, and the test frequency was at OCP with an amplitude of 10 mV. For potentiodynamic polarization (PDP) testing, the scan range was −1000 mV to 1000 mV (vs. OCP), and the scan rate was set at 0.5 mV/s.

### 2.4. Immersion Corrosion Experiment

The sample of immersion corrosion test was prepared by the method of electric spark cutting. The sampling position was the middle of the rolled plate, and the size was 30 × 20 × 2 mm. The immersion corrosion solution selected was a 1 mol/L HCl solution. The specimens were suspended in a beaker and placed in a constant temperature water bath. The temperature was controlled at 25 ± 1 °C using a CS501-3C constant temperature water bath heater. Samples were taken after immersing for 2, 4, 6, 8, and 10 days and sequentially cleaned using ultrasound in acetone, deionized water and alcohol. Subsequently, they were air-dried and weighed on electronic balance with an accuracy of 0.0001 g. The corrosion rate (R) was calculated based on the change in specimen mass. Three parallel specimens were set for each alloy composition, and the average corrosion weight loss data were taken as R.

## 3. Results and Discussion

### 3.1. Microstructure

**Figure 1:**
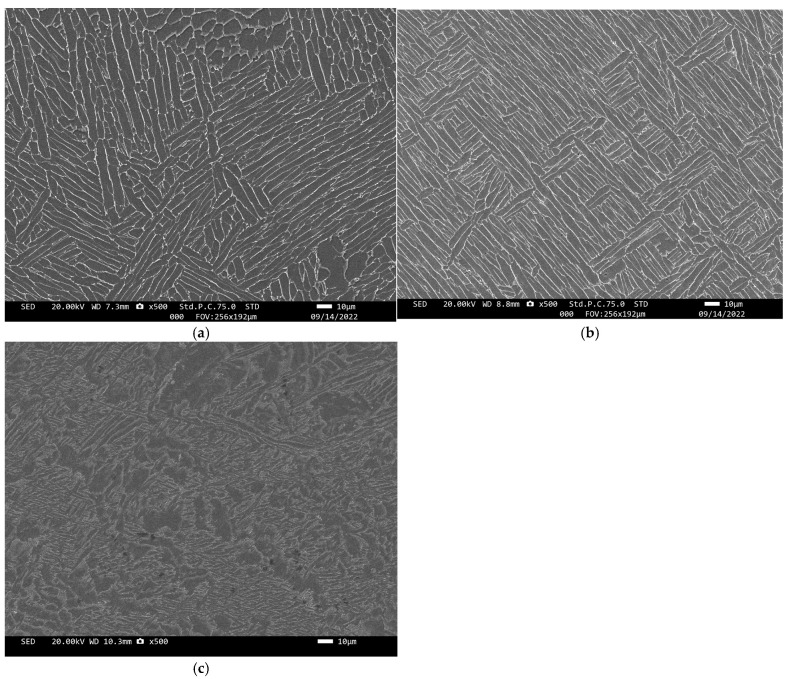
The SEM image of TC4-xNi-yNb alloys’ microstructure: (**a**) TC4-0.5Ni; (**b**) TC4-0.5Nb; (**c**) TC4-0.5Ni-0.5Nb.

Figure 1 shows the microstructure of TC4-0.5Ni, TC4-0.5Nb and TC4-0.5Ni-0.5Nb alloys. All alloys have a lamellar structure with small β-Ti lamellae between large α-Ti lamellae. It is worth noting that the greater solubility of Ni and Nb in β-Ti, as well as the increased stability of β-Ti at room temperature, is responsible for the presence of some β-transformed tissues in TC4-0.5Ni-0.5Nb alloys [12].

### 3.2. Electrochemical Behavior

#### 3.2.1. OCP Tests

**Figure 2:**
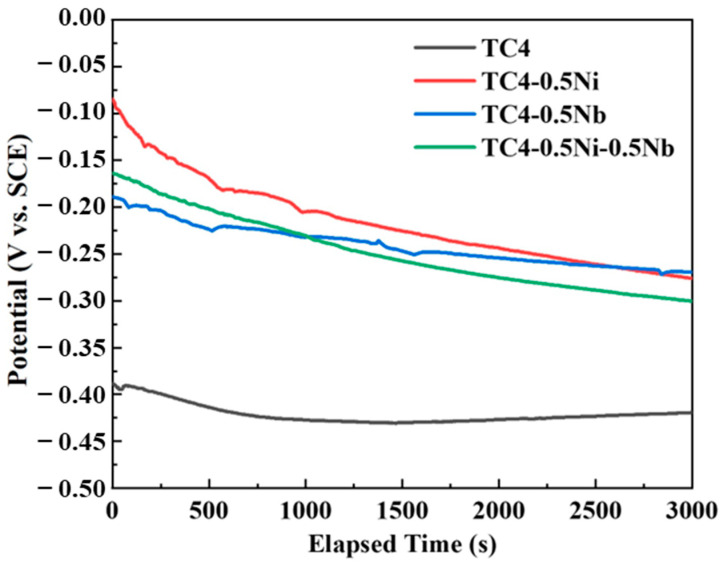
Variation in OCP with immersion time of four alloys in a 1 mol/L HCl solution.

The dissolution and formation patterns of the passivation film of the alloy in the corrosive solution could be analyzed by observing the trend of changes in OCP [19,20]. Figure 2 displays curves showing the variation of OCP with immersion time of four alloys in a 1 mol/L HCl solution. There was a noticeable fluctuation in OCP in the initial stages of immersion, exhibiting a gradual negative trend. The OCP for all alloys tended to stabilize as the immersion time increases. In the initial stages of immersion, the stability of the passivation film on the alloy surface was relatively poor, making it prone to dissolution in the corrosive medium. However, new passivation films would gradually form due to the excellent self-healing capability of the passivation film on titanium alloys. The system eventually reached a balance between the dissolution and generation of passivation films with the extension of immersion time. The final quasi-steady OCP of the TC4 alloy was the lowest, stabilizing around −420 mV vs. SCE in Figure 1. The final OCP values for the other three alloys were significantly higher than that of the TC4 alloy, stabilizing at approximately −300 mV vs. SCE. This difference indicated that the addition of Ni and Nb elements improved the stability of the passivation film.

#### 3.2.2. PDP Tests

**Figure 3:**
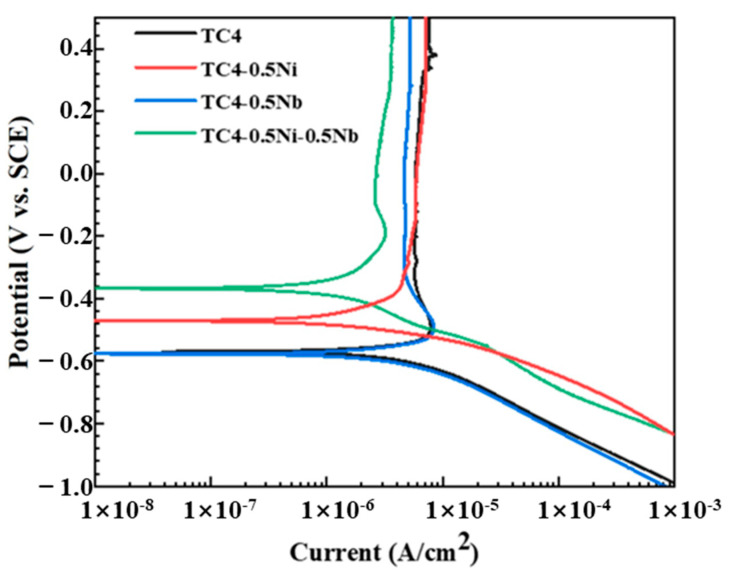
The PDP curves of four alloys in a 1 mol/L HCl solution.

The PDP test was a common technique in electrochemical research which was used to assess the corrosion resistance of materials and understand their electrochemical behavior at different potentials. The PDP curves of four alloys in a 1 mol/L HCl solution were shown in Figure 3. It could be observed that the PDP curves all exhibited similar characteristics, showing cathodic polarization, anodic polarization, activation–passivation and stable passivation regions. The reaction processes corresponding to anodic polarization and activation-passivation regions are as follows [19,21].

Anodic polarization:

(1) $$Ti+H_{2}O↔Ti{\left(H_{2}O\right)}_{ads}$$

(2) $$Ti{\left(H_{2}O\right)}_{ads}↔Ti{\left(OH^{-}\right)}_{ads}+H^{+}$$

(3) $$Ti{\left(OH^{-}\right)}_{ads}↔{\left(TiOH\right)}_{ads}+e^{-}$$

(4) $${\left(TiOH\right)}_{ads}↔{\left(TiOH\right)}_{ads}^{+}+e^{-}$$

(5) $${\left(TiOH\right)}_{ads}^{+}↔{\left(TiOH\right)}_{ads}^{2+}+e^{-}$$

(6) $${\left(TiOH\right)}^{2+}+H^{+}↔{Ti}^{3+}+H_{2}O$$

Activation–passivation:

(7) $${\left(TiOH\right)}_{ads}^{2+}+H_{2}O↔{\left[Ti{\left(OH\right)}_{2}\right]}_{ads}^{2+}+H^{+}+e^{-}$$

(8) $${\left[Ti{\left(OH\right)}_{2}\right]}_{ads}^{2+}↔TiO_{2}+H^{+}$$

The current density initially increased rapidly as the potential increases in the anodic branch indicating active dissolution behavior of the alloy as the anode [8,19]. As the potential continued to increase, the increase in current density was inhibited. Alloys had distinct activation–passivation behavior, manifested by a relatively stable current density with increasing potential.

Indicators were obtained by PDP tests such as the self-corrosion potential (E_corr_), self-corrosion current density (I_corr_), passivation potential (E_pp_) and passivation current density (I_pp_), which were used to evaluate the corrosion resistance performance of metallic materials. Table 2 listed the corrosion kinetic parameters of four alloys obtained through the Tafel extrapolation method in 1 mol/L HCl solution for a clear comparison of the electrochemical corrosion performance among different alloys. TC4 and TC4-0.5Nb exhibited a lower E_corr_ and higher I_corr_ compared to the other two alloys. E_corr_ correlated with how easily a material undergoes corrosion, while I_corr_ was associated with the rate of corrosion dissolution of the material. Therefore, TC4 and TC4-0.5Nb alloys exhibited the highest corrosion susceptibility and a higher corrosion dissolution rate, indicating the poorest corrosion resistance performance. The other two alloys containing Ni exhibited lower I_corr_ and higher E_corr_, demonstrating a lower corrosion susceptibility and higher corrosion resistance performance. The standard electrode potential for Ni was −0.257 V vs. SCE, significantly higher than the standard electrode potentials of Ti at −1.63 V and Nb at −1.099 V with reference to the standard electrode potentials of metals. The addition of Ni played a role in increasing the electrode potential of the alloy matrix reducing the alloys’ susceptibility to corrosion.

I_pp_ represents the current density at which the passivation film dissolved. The lower the I_pp_, the higher the stability of the alloys’ passivation film, indicating a stronger protective capability for the substrate. The initiation potential of entering the stable passivation region on the polarization curve was chosen as the E_pp_ in this article. The current density at this potential was considered as the I_pp_. As shown in Table 2, the addition of Ni and Nb elements led to a varying degree of reduction in the I_pp_ of the alloys with the effect of Nb being more pronounced. Research indicated that the oxide Nb_2_O_5_ formed by the Nb element in the passivation film on the alloy surface could stabilize the passivation film and inhibit the chemical dissolution of titanium alloy in reducing acids [22]. However, the I_pp_ of the alloy did not exhibit a linear decrease with the increase in Nb element, which may be related to the inhibition of TiO_2_ grain growth by Nb^5+^, leading to a reduction in passivation film thickness [15].

#### 3.2.3. Electrochemical Impedance Spectroscopy (EIS)

**Figure 4:**
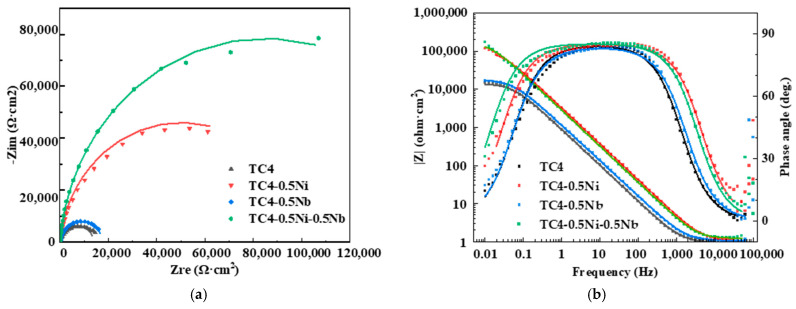
EIS test results of four alloys in 1 mol/L HCl solution: (**a**) Nyquist plot; (**b**) Bode plot.

**Figure 5:**
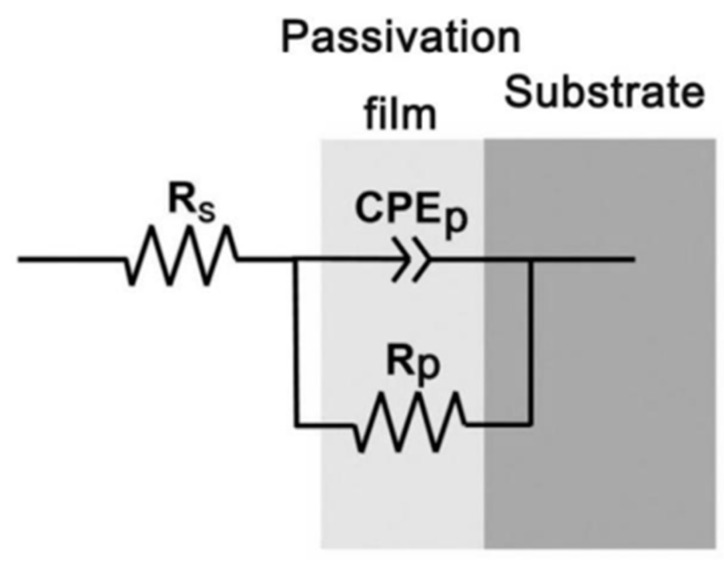
The equivalent circuit diagram for impedance spectrum analysis of titanium alloys.

In order to further analyze the mechanism of the impact of Ni and Nb element additions on the corrosion resistance performance of the alloys, EIS was measured for the four alloys in a 1 mol/L HCl solution and the corresponding Nyquist and Bode plots were obtained as shown in Figure 4. The Nyquist plot (Figure 4a) represented the characteristics of the imaginary and real parts of impedance as a function of frequency. And Figure 5 is the equivalent circuit diagram used for EIS analysis. Bode magnitude and Bode phase angle plots are shown in the Bode plot (Figure 4b), representing the trends of the absolute value and phase angle of impedance as a function of frequency, respectively. The Nyquist plots of the four alloys all exhibit single capacitive loops of different sizes in Figure 4a. TC4 and TC4-0.5Nb alloys had similar impedance arc sizes, significantly smaller than the impedance arcs of the other two alloys. Generally, the smaller the size of the impedance arc, the lower the impedance for charge transfer, indicating poorer corrosion resistance of the material [21,23]. Therefore, the TC4-0.5Ni-0.5Nb alloy exhibited the highest corrosion resistance, followed by the TC4-0.5Ni alloy. In Figure 4b, the phase angles of the four alloys showed only one distinct plateau, indicating that the impedance of the alloys had only one time constant, exhibiting typical capacitive characteristics. The impedance of TC4 and TC4-0.5Nb alloys in the low-frequency range was significantly lower than the other two alloys, indicating that the corrosion resistance of these two alloys was relatively poor.

The EIS results for the four alloys all conformed to the most commonly used compact passivation film equivalent circuit model. The simplified Randles equivalent circuit was selected to fit the data [24]. The equivalent circuit diagram for impedance spectrum analysis of titanium alloys is shown in Figure 5. Rs represented the solution resistance between the reference electrode and the working electrode, while Rp represented the polarization resistance at the interface between the working electrode and the solution. CPE was a constant phase angle element representing the capacitance of the passivation film. CPE was introduced to describe the non-ideal behavior of the capacitance element, arising from the surface unevenness and non-uniform current distribution caused by electrode surface roughness, impurities, dislocations or grain boundaries [12]. CPE was represented by CPE_T_ and CPE_P_, where CPE_T_ was the film capacitance and CPE_P_ was the deviation parameter. When CPE_P_ = 1, CPE represented a pure capacitor. A smaller CPE_P_ value indicated a greater deviation of CPE from an ideal capacitor, suggesting more defects in the passivation film on the alloy surface. The polarization resistance R_p_ was directly proportional to the corrosion resistance of the alloy. Numerical fitting of the equivalent circuit in Figure 4 was performed using Zview 2.0 software, and the obtained parameters are shown in Table 3. The χ^2^ values of all materials are about 10^−3^, which indicates that the fitting results is credible.

The R_p_ fitted for TC4 and TC4-0.5Nb was similar, significantly lower than the other two alloys, indicating that TC4 and TC4-0.5Nb alloys had the poorer corrosion resistance. On the other hand, the alloy TC4-0.5Ni-0.5Nb exhibited the highest fitted R_p_, demonstrating the best corrosion resistance, consistent with the conclusions drawn from the PDP.

The thickness of the passivation film could, to some extent, reflect the corrosion resistance of the alloy. Wang et al. estimated the thickness of the passive film based on the EIS fitting experimental data as shown in Formula (9) [25]:

(9) $$d=\frac{\varepsilon \delta S}{C}$$

where d represents the passivation film thickness fitted through EIS data; C denotes the passivation film capacitance with the numerical value being the CPET value; ε stands for the dielectric constant of the passivation film, typically set to ε = 100 for titanium alloys; δ represents the vacuum dielectric constant with δ = 8.86 × 10^−12^ F/m; and S is the surface area of the test specimens.

**Figure 6:**
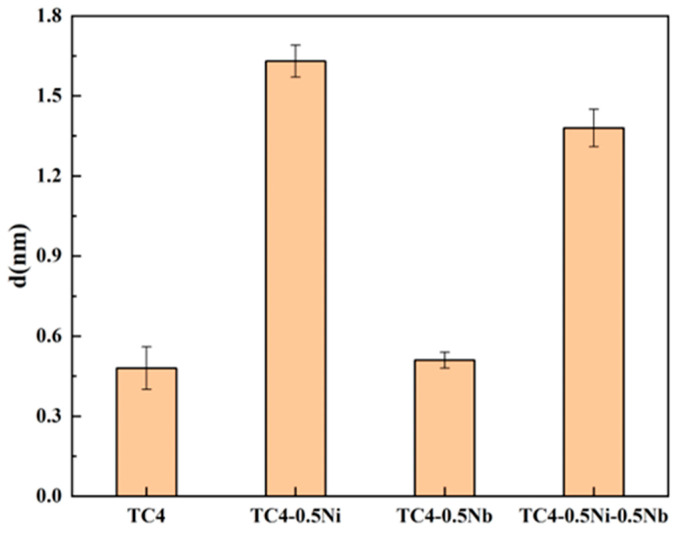
Passivation film thicknesses of four alloys in a 1 mol/L HCl solution.

The passivation film thicknesses of four alloys obtained through EIS measurements are depicted in Figure 6. Passivation film became more effective in protecting the substrate as their thickens, resulting in higher corrosion resistance of the alloy. The passivation film thicknesses of TC4-0.5Ni and TC4-0.5Ni-0.5Nb in Figure 6 are significantly higher than that of TC4 and TC4-0.5Nb alloys. This confirms that the addition of Ni elements significantly improved the corrosion resistance of the alloy. Combining the electrochemical experimental data above, although the passivation film thickness of the TC4-0.5Ni-0.5Nb alloy was slightly thinner than that of the TC4-0.5Ni alloy, it still exhibited higher corrosion resistance, which may be attributed to the stabilizing effect of the oxide formed by Nb elements on the passivation film at the alloy surface.

### 3.3. Corrosion Weight Loss

The corrosion weight loss experiment evaluated the corrosion resistance of materials by measuring the difference in the material’s initial mass and its mass at the end of the experiment. The corrosion weight loss rate was calculated using Formula (10) [19]:

(10) $$R=\frac{8.76\times 10^{7}\times M}{\mathrm{STD}}$$

where R represents the annual corrosion rate of the alloy (mm/a); M represents the mass loss of the sample during immersion (g); S is the surface area of the immersed corroded sample (cm^2^); T is the immersion time for corrosion (h); and D is the density of the tested sample (kg/m^3^).

**Figure 7:**
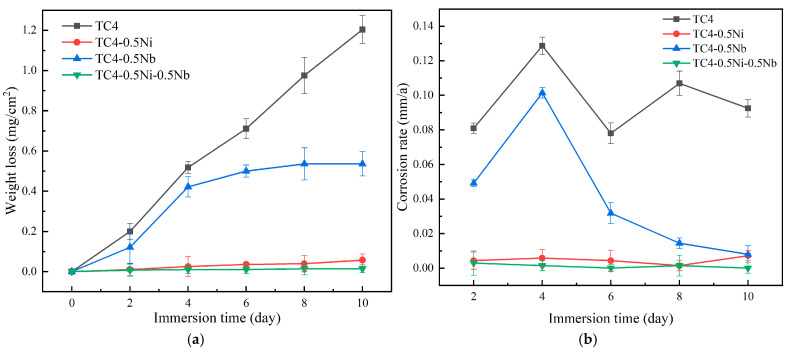
Corrosion weight loss curves and corrosion rate plots of the four alloys immersed in a 1 mol/L HCl solution for ten days: (**a**) alloy corrosion weight loss curves; (**b**) variation curves of corrosion rates.

Figure 7 depicts the corrosion weight loss data and the variation curves of corrosion rates of the four alloys in a 1 mol/L HCl solution as the immersion time. In the early stages of immersion, both TC4 and TC4-0.5Nb alloys exhibited significant corrosion weight loss and weight loss rates, showing a trend of gradual increase in corroded mass loss with prolonged immersion time. The corrosion rates of both alloys gradually decreased and tended to stabilize with the extension of immersion time. In the later stages of immersion, the corrosion rate of TC4-0.5Nb alloy was significantly lower than that of TC4 alloy. In the early stages of corrosion, the passivation films on these two alloys were less stable. As corrosion progresses, the formation and dissolution of passivation films of TC4 alloy reached a dynamic equilibrium, resulting in a linear increase in corrosion weight loss. Meanwhile, the oxide of Nb played a stabilizing role in the newly formed passivation film on the alloy surface and enhanced the corrosion resistance of the passivation film. As a result, the dissolution rate of TC4-0.5Nb alloy significantly decreased in the later stages of the immersion phase. The addition of Ni significantly reduced the corrosion weight loss of the alloy with an annual average corrosion rate not exceeding 0.001 mm/a, belonging to the category of mild corrosion. Specifically, the corrosion rate of TC4-0.5Ni-0.5Nb was essentially 0 mm/a, demonstrating the highest corrosion resistance performance. This result was consistent with the findings from electrochemical experiments.

**Figure 8:**
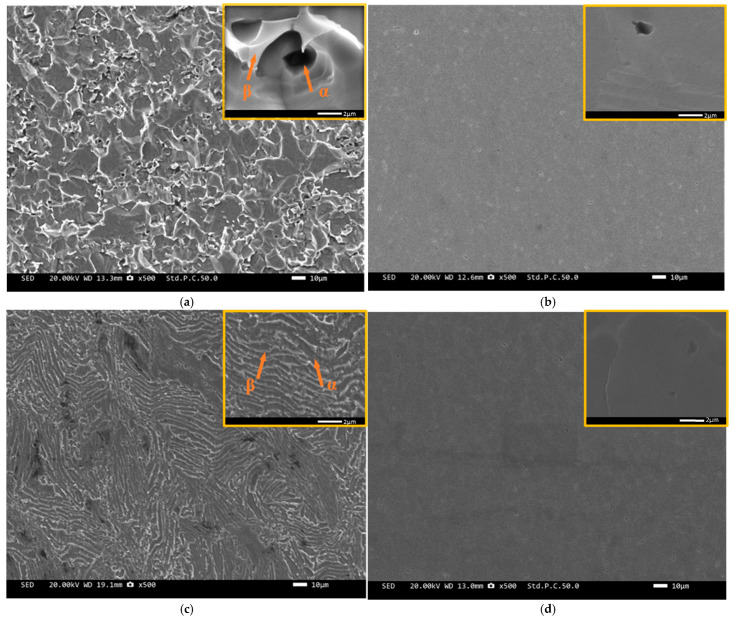
The corrosion morphology observation of the four alloys after ten days of immersion in a 1 mol/L HCl solution: (**a**) TC4; (**b**) TC4-0.5Ni; (**c**) TC4-0.5Nb; (**d**) TC4-0.5Ni-0.5Nb.

Figure 8 shows the surface corrosion morphology of the four alloys after immersion in a 1 mol/L HCl solution for ten days. The α phase of TC4 alloy underwent severe corrosion in 1 mol/L HCl solution, as shown in Figure 8a, that may be attributed to the potential difference between the α and β phases, leading to the formation of micro-galvanic cells and causing localized corrosion [19,26,27]. The corrosion of TC4-0.5Nb alloy was relatively mild, and it also exhibited preferential corrosion of the lamellar α phase, as shown in Figure 8c. As shown in Figure 8b,d, the corrosion morphology of the alloys was similar with all exhibiting grain boundary corrosion and pitting corrosion after the addition of Ni elements. Atoms had higher activity and more defects at grain boundaries, making them more susceptible to corrosion. Comparing Figure 8b,d, the pitting corrosion on the surface of TC4-0.5Ni alloy was larger and deeper than that on TC4-0.5Ni-0.5Nb alloy. Additionally, TC4-0.5Ni alloy had a greater number of pitting corrosion sites. This indicated that TC4-0.5Ni-0.5Nb had the best corrosion resistance performance, combining the electrochemical and corrosion weight loss test data.

### 3.4. Corrosion Resistance Mechanism

The excellent corrosion resistance of titanium and its alloys primarily depends on the stability of the surface passivation film. Studies have shown that TiO_2_ passivation film can effectively isolate the corrosive medium from the titanium alloy matrix and reduce the reaction between them, thereby lowering the corrosion dissolution rate of the alloy [28,29,30]. An analysis of the passivation film on the alloy surface at different immersion corrosion times was conducted to investigate the evolution of the passivation film on the alloys as the immersion corrosion time extends.

**Figure 9:**
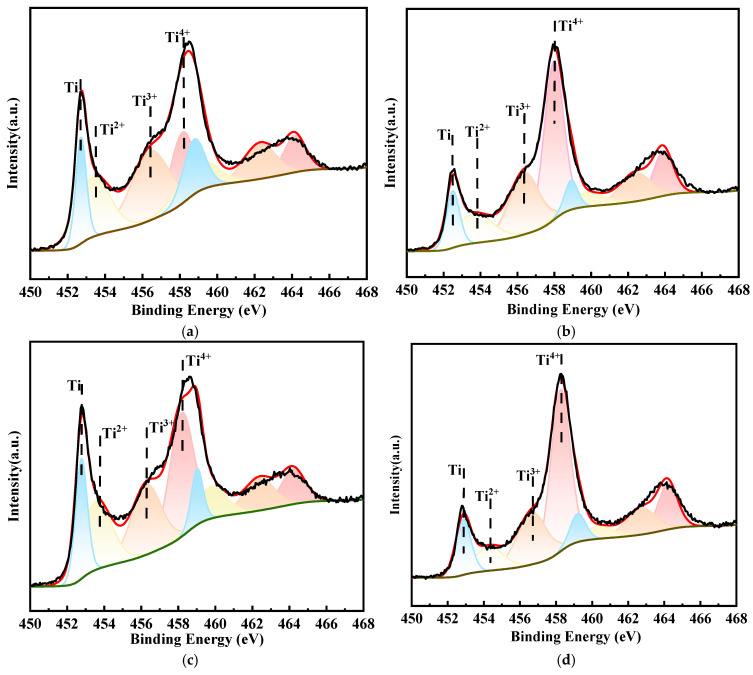
Narrow-scan fine spectra of Ti2p on the surface of the four alloys in the initial state: (**a**) TC4; (**b**) TC4-0.5Ni; (**c**) TC4-0.5Nb; (**d**) TC4-0.5Ni-0.5Nb.

Figure 9 shows the Ti_2p_ narrow scan spectra of the surfaces of the four alloys not in contact with the corrosive medium. The Ti_2p_ fine spectrum consisted of eight small peaks corresponding to four valence states: Ti^4+^_2p3/2_, Ti^3+^_2p3/2_, Ti^2+^_2p3/2_ and Ti_2p3/2_. These peaks represented four chemical components: TiO_2_, Ti_2_O_3_, TiO and elemental Ti [24,27,31]. The Ti elements on the surface of the passive film of the four alloys existed in the form of Ti^4+^ before coming into contact with the corrosive medium, indicating that the main structure of the passive film on the alloy surface was TiO_2_, and it contained a certain amount of Ti^2+^, Ti^3+^ and Ti. The ratios of various ions in the passivation film calculated based on the peak areas corresponding to the valence states of Ti are shown in Table 4. The passivation films on TC4 and TC4-0.5Nb alloys indicated a lower content of Ti^4+^, accounting for 20% and 30%, respectively. They also contained a larger proportion of Ti^2+^ and Ti^3+^, indicating that the contents of metastable oxides TiO and Ti_2_O_3_ in the passivation films of these two alloys were high. Research indicated that Ti^4+^ in TiO_2_ could not undergo further oxidation and was more stable compared to metastable oxides like Ti_2_O_3_ and TiO_2_ [32]. In this scenario, Ti^2+^ and Ti^3+^, serving as defects in the passivation film, could damage the integrity of the passivation film. Therefore, the lower the contents of Ti^2+^ and Ti^3+^, the better the stability of the passivation film. It also indicated that the higher corrosion rates of TC4 and TC4-0.5Nb were primarily due to the dissolution of the unstable passivation film on the surface during the initial immersion period.

**Figure 10:**
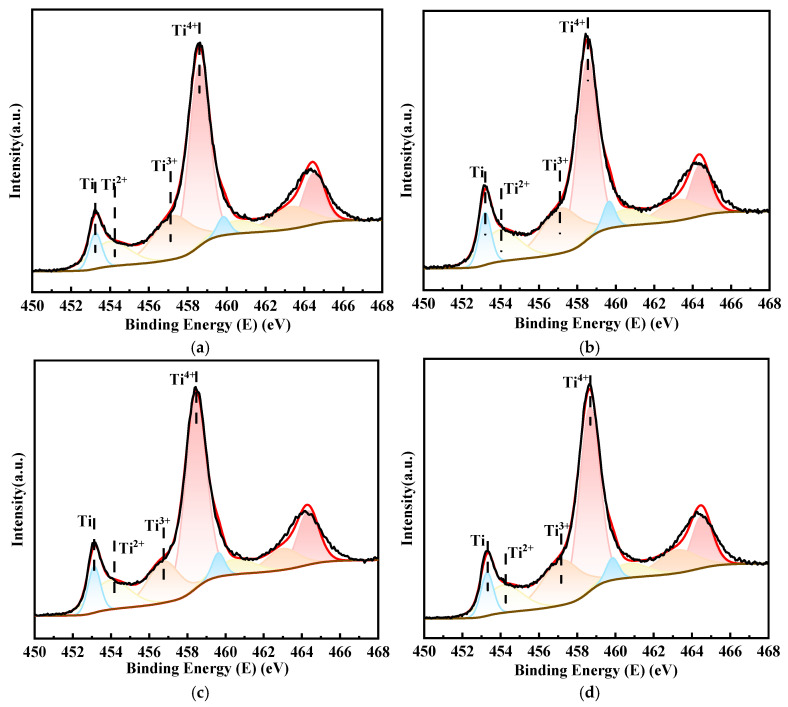
Ti2p narrow-scan fine spectra on the surface of four alloys after immersing in a 1 mol/L HCl solution for ten days: (**a**) TC4; (**b**) TC4-0.5Ni; (**c**) TC4-0.5Nb; (**d**) TC4-0.5Ni-0.5Nb.

Figure 10 shows the Ti_2p_ narrow-scan spectra of the surfaces of the four alloys after immersing in a 1 mol/L HCl solution for ten days. The ion ratios in the passivation film are presented in Table 5. The reduction in the content of Ti^2+^ and Ti^3+^ in the passivation film on the surfaces of TC4 and TC4-0.5Nb alloys suggested an improvement in the stability of the alloy’s passivation film that was also consistent with the subsequent decrease in corrosion rates of the alloys after prolonged immersion. The changes in Ti content for each valence state were minimal in the three alloys with added Ni elements, indicating that the stability of the passivation film on the alloy surface was high.

**Figure 11:**
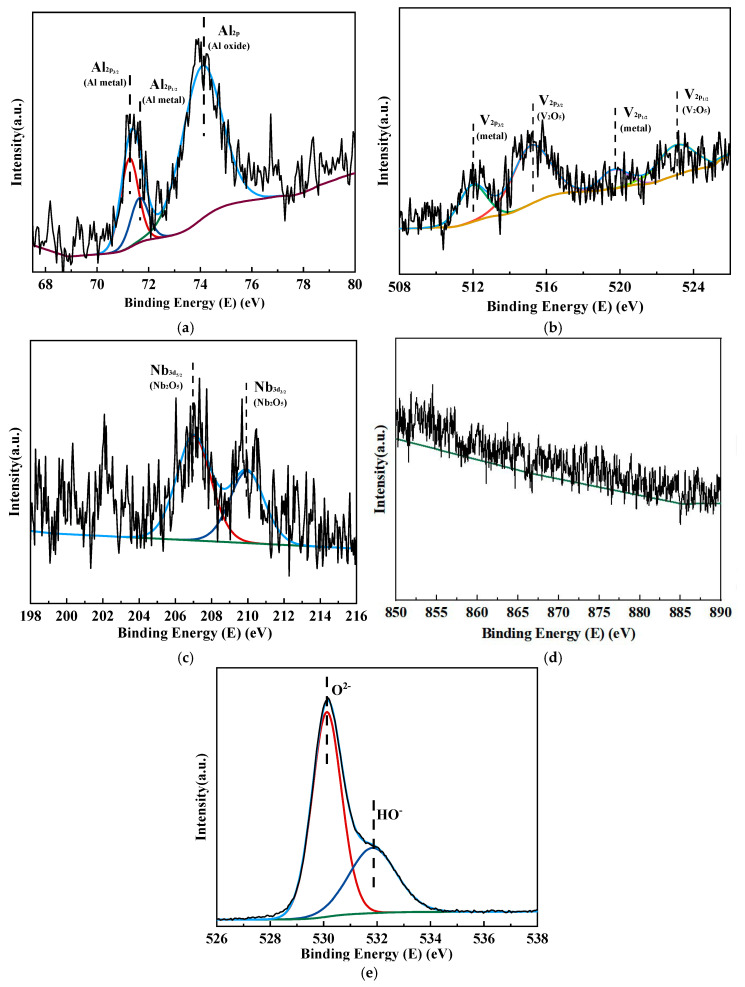
The narrow scan fine spectra of surface elements of TC4-0.5Ni-0.5Nb alloy after ten days of immersion in a 1 mol/L HCl solution: (**a**) Al; (**b**) V; (**c**) Nb; (**d**) Ni; (**e**) O.

Studies indicated that the alloy elements Al, V, Nb, and Ni also formed oxides on the surface of the alloy and had varying effects on the stability and corrosion resistance of the passivation film [32]. Figure 11 shows the narrow scan spectra of each alloy element in the passivation film on the surface of the TC4-0.5Ni-0.5Nb alloy after ten days of immersion in HCl solution. Al primarily existed in the form of Al^3+^ (Al_2_O_3_), while V mainly existed in the form of V^5+^ (V_2_O_5_) in the passivation film on the surface of the TC4-0.5Ni-0.5Nb alloy. Nb was predominantly present in the forms of Nb^5+^ and Nb^4+^ with the content of Nb^5+^ being greater than Nb^4+^, indicating that Nb primarily existed in the form of Nb_2_O_5_ on the alloy surface with a small amount of NbO_2_. Figure 10d showed the narrow scan spectrum of Ni_2p_, but no distinct peaks corresponding to elemental Ni or nickel oxide were observed.

**Figure 12:**
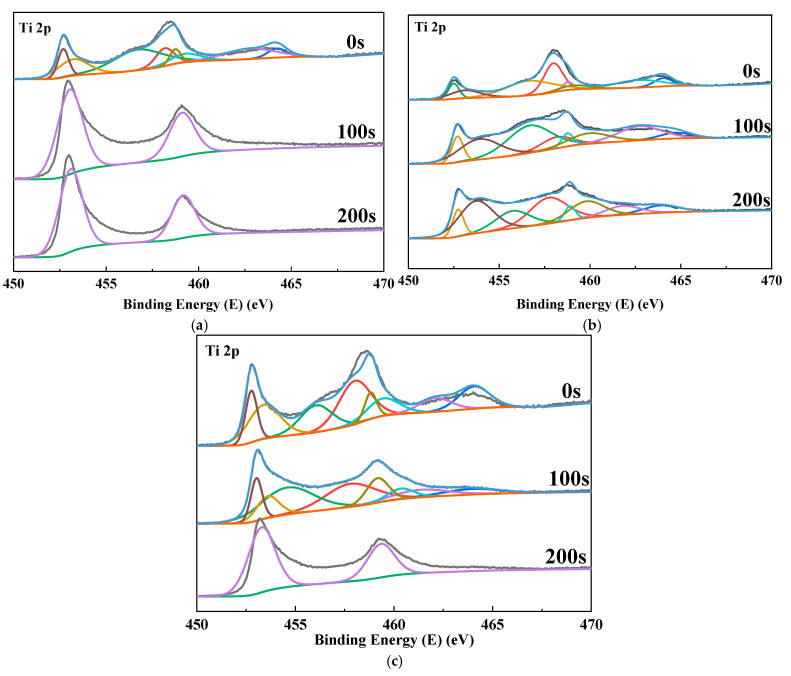
Three alloys’ corrosion and etching results after ten days of immersion are as follows: (**a**) TC4; (**b**) TC4-0.5Ni; (**c**) TC4-0.5Nb.

The passivation film would prevent O from entering the titanium alloy matrix once the thickness and compactness of the passivation film increase to a certain extent. In other words, internal elements within the alloy could no longer react with O, inhibiting further growth of the passivation film. The compositions of the passivation film of the five alloys were analyzed through XPS etching experiments to investigate the changes in the passivation film on the alloy surface after the addition of Ni and Nb elements. In this experiment, the relative sputtering rate of Ar ions to SiO_2_ was 0.05 nm/s. The sputtering times were 0 s, 100 s, and 200 s, respectively. We analyzed the depth sputtering spectra and obtained peak parameters based on signal quantities, as shown in Figure 12. The outermost layer of the three alloys was mainly composed of TiO_2_, with a small amount of low-valence state oxide TiO_x_ (1 ≤ x ≤ 2) present simultaneously. As shown in Figure 12, the TC4 film was primarily composed of elemental Ti after 100 s of sputtering. Additionally, the content of elemental Ti in TC4-0.5Nb was significantly increased. In contrast, for TC4-0.5Ni alloy, a substantial amount of elemental Ti was only detected after a sputtering time of 200 s. In general, the sputtering time corresponding to the significant appearance of elemental Ti or the reduction in O content to zero could be used to estimate the thickness of the titanium alloy film [33]. After calculation, the thickness of the TC4 alloy film was determined to be less than or equal to 5 nm, while the thickness of the TC4-0.5Nb alloy film was approximately 10 nm.

Combining the information from electrochemical studies, immersion corrosion, observations of corrosion morphology and analysis of surface passivation films, it could be observed that the significant improvement in the corrosion resistance of the alloy in a 1 mol/L HCl solution was primarily attributed to the addition of Ni. The addition of Ni enhanced the alloy’s electrode potential and simultaneously increased the stability and thickness of the passivation film, thereby preventing corrosion. The addition of Nb contributed relatively modestly to the enhancement of corrosion resistance in the HCl solution. Nb formed an adherent oxide, Nb_2_O_5_, on the alloy surface passivation film, aiding in maintaining the integrity of the passivation film and improving the alloy’s corrosion resistance. In this context, the synergistic effect of Ni and Nb significantly reduced the alloy’s corrosion susceptibility. This collaboration minimized defects in the surface passivation film and effectively reduces dissolution and cracking of the passivation film, thus enhancing the alloy’s corrosion resistance. The cooperative action resulted in the TC4-0.5Ni-0.5Nb alloy exhibiting optimal corrosion resistance.

## 4. Conclusions

The influence and mechanisms of Ni and Nb element additions on the corrosion resistance of TC4 alloy were explored in this article, leading to the main following conclusions:

1. The addition of Ni elements resulted in high values for self-corrosion potential, polarization resistance and passivation film thickness, along with low self-corrosion current density, showcasing excellent corrosion resistance. The addition of Nb elements could increase the alloy’s passivation current density, but had a minor impact on self-corrosion potential and self-corrosion current density.
2. Corrosion weight loss results indicated that the corrosion rate of the alloy with Ni addition did not exceed 0.001 mm/a. TC4 and TC4-0.5Nb alloys showed significant corrosion weight loss and loss rate in the initial corrosion stage. The corrosion rate of TC4-0.5Nb alloy gradually decreased and tended to stabilize with prolonged immersion time. XPS results suggested this was due to a significant increase in Ti^4+^ content in the newly formed passivation film and enhanced the stability of the alloy passivation film and hindering further corrosion.
3. The passivation film on the alloy surface was primarily composed of TiO_2_, accompanied by the presence of oxides of various elements. The addition of Ni and Nb elements had different degrees of influence on the composition and structure of the alloy passivation film. Ni elements elevated the matrix electrode potential and significantly increasing the thickness of the alloy passivation film. Nb elements existed mainly in oxide form in the passivation film, contributing to the enhancement of the stability of the alloy passivation film.
4. The addition of Ni, an element with high corrosion resistance and easy passivation, improved the thermodynamic stability and passivation film thickness of TC4 titanium alloy. The oxide formed by Nb stabilized the passivation film, enhancing the corrosion resistance of TC4 titanium alloy. Ni and Nb enhance the corrosion resistance of TC4 alloys in different ways. Through optimization, TC4-0.5Ni-0.5Nb alloy exhibited superior corrosion resistance.

## Figures and Tables

**Table 1:** Chemical compositions (wt.%) of four sheets.

Nominal Composition	Al	V	Ni	Nb	Ti
TC4	5.75	3.86	0	0	Bal.
TC4-0.5Ni	6.10	4.06	0.45	0	Bal.
TC4-0.5Nb	5.96	3.99	0	0.58	Bal.
TC4-0.5Ni-0.5Nb	5.87	3.95	0.55	0.56	Bal.

**Table 2:** Corrosion kinetic parameters of four alloys in a 1 mol/L HCl solution.

Nominal Composition	I_corr_ (10^−7^ A/cm^2^)	E_corr_ (mV vs. SCE)	I_pp_ (10^−6^ A/cm^2^)	E_pp_ (mV vs. SCE)
TC4	23.11 ± 5.51	−573.16 ± 13	5.94 ± 0.11	−481.18 ± 12
TC4-0.5Ni	9.14 ± 2.11	−473.85 ± 10	5.13 ± 0.28	−366.82 ± 16
TC4-0.5Nb	21.36 ± 5.13	−564.76 ± 11	4.18 ± 0.16	−336.45 ± 10
TC4-0.5Ni-0.5Nb	6.72 ± 1.07	−370.73 ± 15	3.66 ± 0.31	−270.21 ± 7

**Table 3:** EIS fitting data results of four alloys in a 1 mol/L HCl solution.

Nominal Composition	R_s_ (Ω·cm^2^)	R_p_ (kΩ·cm^2^)	CPE_T_ (Ω^−1^·S^n^·cm^−2^)	CPE_P_	χ^2^ (10^−3^)
TC4	0.960	14.048	0.000181	0.9425	1.63
TC4-0.5Ni	1.105	100.660	0.000055	0.9438	1.57
TC4-0.5Nb	1.063	17.786	0.000169	0.9305	1.31
TC4-0.5Ni-0.5Nb	1.223	170.270	0.000062	0.9482	1.49

**Table 4:** The percentage of Ti elements in different valence states in the passivation film on alloys’ surfaces.

Nominal Composition	Ti	Ti^2+^	Ti^3+^	Ti^4+^
TC4	22.24	26.96	30.26	20.54
TC4-0.5Ni	12.59	17.91	29.46	39.97
TC4-0.5Nb	23.57	21.42	26.98	30.28
TC4-0.5Ni-0.5Nb	15.44	12.9	27.83	43.82

**Table 5:** The percentage of Ti elements in different valence states on the surface passivation film of the five alloys after immersing in a 1 mol/L HCl solution for ten days.

Nominal Composition	Ti	Ti^2+^	Ti^3+^	Ti^4+^
TC4	7.41	16.43	28.69	47.48
TC4-0.5Ni	9.80	19.54	26.11	44.53
TC4-0.5Nb	9.42	18.57	21.52	50.49
TC4-0.5Ni-0.5Nb	8.62	17.15	28.03	46.21

## Data Availability

The data presented in this study are available on request from the corresponding author. The data are not publicly available due to the authors do not have permission to share data.
